# Ferritin as a predictive salivary marker for ECC-III

**DOI:** 10.6026/973206300213876

**Published:** 2025-10-31

**Authors:** Kinjal Vekariya, Shantanu Choudhari, Gadhiya Niraliben, Karishma Desai, Shivani Shah, Priya Amin

**Affiliations:** 1 Department of Pediatric Dentistry, Government Dental College and Hospital, Ahmedabad, India; 2 Department of Dental Surgery, Government Dental College and Hospital, Ahmedabad, India; 3 Department of Dental Surgery, P.M.N.M. Dental College and Hospital, Karnataka, India

**Keywords:** Early childhood caries (ECC), salivary ferritin, salivary biomarker, saliva

## Abstract

Dental caries is a prevalent disease with a significant impact on children. Saliva, containing various organic and inorganic substances, may serve as a biomarker for identifying dental caries. Therefore, it is of interest to evaluate the correlation between salivary ferriti levels and ECC-III in 60 children aged 4-5 years, divided into a caries-free control group and an ECC-III group (≥5 decayed, absent, or filled teeth). Salivary ferritin levels were measured using a diagnostic kit, and statistical analysis showed significantly higher levels in the ECC-III group compared to controls (p < 0.0001). Thus, we show salivary ferritin may serve as a simple, non-invasive biomarker for assessing severity and predicting progression of ECC.

## Background:

Saliva, containing a variety of organic and inorganic elements, presents itself as a promising indicator for detecting dental cavities, providing clinicians with valuable understanding of the infection process and aiding in making treatment decisions based on evidence, by using salivary kits. The consistent capacity to identify biochemical markers is facilitating the advancement of biomedical research through the examination of a variety of bodily secretions. Saliva stands out as a crucial diagnostic fluid, offering notable biochemical and practical benefits over blood [1, 2]. Saliva serves a crucial function in preserving the health of dental structures by containing essential minerals like calcium and phosphorus, which actively contribute to the remineralization process, offering a natural defence mechanism against tooth decay and dissolution. Saliva's role in maintaining dental tissue integrity is underscored by its content of calcium, phosphorous, and other inorganic ions, which promote the remineralization of early enamel lesions or demineralized areas, thereby forming a natural defence mechanism against tooth dissolution [3]. Researchers propose that molecules from the blood stream entering salivary tissues via different routes could potentially alter the composition of oral fluids, suggesting that disease biomarkers absorbed by the salivary glands may convey information about an individual's health status. Early childhood caries (ECC) diagnosis and prevention is crucial in order to advance favourable future oral health outcomes is emphasized by the robust association between ECC and the eventual development of caries in both permanent and primary teeth. Saliva and blood are key fluids in clinical diagnostics; saliva's painless collection, ease of use, and storage convenience make it preferable over serum, allowing multiple collections from one individual without specialized training [4, 5, 6-7]. Examining ferritin levels in saliva provides a non-invasive alternative to venous blood draws, traditionally used to detect iron deficiency anaemia, especially in pediatric patients, addressing concerns of contamination and risk associated with invasive procedures, while potentially serving as a reliable predictor of the disease's onset, significance of salivary ferritin levels in early detection and surveillance is underscored by the fact that they may occur prior to haematological changes. Further, structural, histochemical, and clinical changes occur before significant red cell morphology and haemoglobin levels are altered [8]. Therefore, it is of interest to determine the salivary Ferritin levels in children with iron deficiency anaemia and assess its reliability as a predictive marker of the disease. The null hypothesis was that there is no alteration of salivary ferritin levels in children with iron deficiency anaemia.

## Materials and Methods:

## Study design:

This cross-sectional observational study was attempted to determine the connection among early childhood caries and salivary ferritin levels. The study group and control group consisted of 30 children each respectively. Thirty boys and thirty girls between the ages of four and five were included in this investigation. The study protocol was approved by the Institutional Ethical Committee (No. IEC GDCH/PED.3/2021). Prior to the collection of saliva, written authorization was acquired from the parent or guardian for all individuals below the age of 18. Demographic information and medical history were acquired subsequent to the participant's consent to participate in this investigation. The principal investigator conducted an intraoral examination under appropriate illumination using a dental explorer and mouth mirror.

## Inclusion and exclusion criteria:

The study included healthy children between the ages of four and five who underwent either a dental check- up or dental treatment at the Department of Pediatric and Preventive Dentistry. The study group consisted of children with ECC-III (decayed, absent, or filled >/=5) who never had any dental treatment. Children who were age-matched with the study group and had been free of caries were included in the control group. Children who were medically compromised, not willing to provide parental consent, and consuming any medications that could potentially affect ferritin levels were excluded from this study.

## Methods:

Subsequent visits were scheduled for participants who were eligible to participate. They were advised to brush their teeth and consume prior to the collection of the salivary sample to guarantee the quality for the analysis. In the second visit, a 5 mL syringe was used to collect 1-5 mL of unstimulated whole saliva from the floor of the mouth using the vacuum method. The collection of saliva samples took place between the hours of 9:00 a.m. and 12:00 p.m. The saliva was refrigerated at a temperature of 2-8 in a container that is clearly labelled by the subject's identification number (serial number). The samples were then taken to the (ICMR- NIOH) refers to the Institute of Occupational Health from the Department of Medical Research in the biochemistry laboratory in Ahmedabad, Gujarat, India, for the salivary ferritin assay, using a refrigerated storage box. The commercially available autozyme diagnostic kit (ClinReact ITA Ferritin Test Reagent kit, Accurex Biomedical Pvt. Ltd., Mumbai, India) was employed to determine S-Ferritin levels. The samples were analysed in accordance with the kit's instructions, and the salivary ferritin level was calculated using the Gen 5 version 2.04 software Ferritin assay for all of the subject's samples. The concentration of salivary ferritin was recorded in a (MS Office 2019) Microsoft Excel worksheet and the entire dataset was transmitted for statistical analysis.

## Results:

Salivary ferritin values were analysed using the All-In-One Microplate Reader Software, Gen 5 version 2.04. The Mean Salivary Ferritin was correlated between two groups using an unpaired "t" test. The mean salivary ferritin and dmft were correlated using the ANOVA test. Salivary ferritin and dmft were correlated with the gender of participants using an unpaired "t" test. A P value of 0.05 or lower was considered to be significant in this study. (X ± SD) The results are presented in the form of the mean plus the standard deviation (SD). Table 1 (see PDF) shows salivary ferritin values in the control and study groups. There is a statistically highly significant difference noted in salivary ferritin level between the study group (151.21 ± 26.33ng/dl) and control group (85.78±12.52ng/dl). On applying the unpaired "t" test, the two groups shows a statistically highly significant difference in salivary ferritin levels, as p value is <0.0001. Table 2 (see PDF) shows association of salivary ferritin with mean dmft score in study and control group. There is a significant increase noted in salivary ferritin level on increasing dmft score (dmft score 0 - 85.78ng/dl, dmft score 5-10 - 139.43ng/dl, dmft score 11- 15 - 7.13ng/dl, dmft score >15 189.48ng/dl). On applying ANOVA Test, p value is 0.0*, which shows statistically highly significant association between salivary ferritin and increased dmft score. Table 3 (see PDF) shows the association of gender and dmft score. There is no significant difference noted in mean dmft score between males and females (Male- 157.53, Female- 143.98). On applying unpaired "t" test p value is 0.16 that is statistically not significant. This shows that there is no association between caries experience.

## Discussion:

Most developing countries are currently experiencing a rising prevalence of dental caries. Dental caries, also referred to as tooth decay, is a highly common chronic ailment affecting people globally. Individuals remain vulnerable to this condition throughout their entire lifespan. ECC (Early childhood caries) can manifest as a severe form of tooth decay that specifically impacts the primary teeth of toddlers. ECC's relationship with numerous health conditions, including as impoverishment, gastrointestinal illnesses, discomfort, and insomnia has elevated it to the status of a significant public health concern. Jagannathan *et al.* found that salivary ferritin can serve as a diagnostic biomarker for identifying iron deficiency anaemia. Similar to how Lin-na Guo *et al.* demonstrated, salivary ferritin can be used to diagnose both type II diabetes mellitus and iron deficiency anaemia. Furthermore, they demonstrated that assessing salivary ferritin levels could serve as a baseline for calculating the quantity of iron in the body [9, 10]. Salivary ferritin was utilized in place of GCF-ferritin in this investigation due to its superior ease of collection in large quantities and it's significantly less invasive nature when contrasted with other body fluids, including gingival crevicular fluid and serum. The vast majority of the analytical chemicals detected in serum from the blood are additionally found in saliva, although at much lower concentrations. Nevertheless, the association between salivary and blood-based substances suggests that, despite their distinct and dissimilar natures, they may be connected at the molecular level. Systemic conditions are monitored by a plethora of biological markers that are present in human saliva. The suction method was employed in saliva collection due to its ability to acquire complete saliva without the need for stimulation. The saliva of every participant was obtained between 9 a.m. and 12 p.m., as the human body's circadian rhythm indicates that saliva flow is at its highest during this time [11, 12]. The correlation between salivary ferritin and early childhood caries has been examined in the present study, as it is suspected that they are linked to each other and iron deficiency anaemia. ECC is a risk factor for anaemia, as concluded by Hashemi *et al.* This connection between two significant public health issues may have been caused by dental pain, which may have restricted dietary consumption. Iron deficiency has been recognized as a risk factor for ECC in numerous studies. The precise cause of this increase in salivary ferritin is unknown. One possible explanation for this increase is because ferritin, an iron-dependent enzymatic function of saliva, is being preserved. However, ferritin endocytosis and subsequent excretion via the salivary ducts is another possible explanation. Another possible explanation for the rise in salivary ferritin could be the presence of large-molecular-weight iron-binding proteins or the uptake of ferritin through the intercalated ducts of the parotid [13, 14, 15, 16- 17]. In the present study Ferritin in the saliva is present in both control and study groups with mean 85.78ng/ml (SD 12.52) and 151.21(SD 26.33) when comparing the caries-active group to the caries-free group, were discovered to be considerably greater in the later, P value of <0.0001. The present study showed that salivary ferritin levels increased progressively with higher dmft scores. Statistical analysis revealed a significant correlation between ferritin concentration and caries severity, indicating that salivary ferritin may reflect the extent of dental caries activity [18, 19-20]. The statistical analysis in this study found negative significant variation in salivary ferritin content between males and females. The data from this study can potentially help clinicians using non-invasive methods to measure ferritin level in saliva which is correlated with serum ferritin and iron load of the body so, iron deficiency anaemia can be diagnosed at early stage and by non-invasive method especially in pediatric patients and its future consequences can be prevented at early stage. Caries in the teeth might be an indicator of low iron levels, so it's important to treat them before they worsen the level of iron and deficiency occurs. Iron and haemoglobin levels should be evaluated before surgery on patients with severe early childhood caries, as iron performs an essential function in optimal physical and neurological development. An additional potential mechanism is the elevated levels of salivary manganese, which can impede the transport of salivary ferritin, resulting in its retention. This leads to an elevated ferritin level in the saliva of people with iron deficient anaemia. Nevertheless, the limited literature on this subject prevents us from establishing a precise pathogenesis for the increase in ferritin.

## Conclusion:

Based on the findings of our analysis, we have determined that there is an unambiguous relationship between salivary ferritin and dmft. Children with high dmft scores exhibited a higher salivary ferritin level, and there is no correlation between gender and caries experience.

## Additional Information:

## Disclosures:

## Human subjects:

Consent was obtained or waived by all participants in this study. Institutional Ethics Committee, Government Dental College and Hospital, Ahmedabad issued approval IEC GDCH/ PED.3/2021. The study was approved by Institutional Ethics Committee, Government Dental College and Hospital, Ahmedabad.

## Animal subjects:

All authors have confirmed that this study did not involve animal subjects or tissue.

## Financial relationships:

All authors have declared that they have no financial relationships at present or within the previous three years with any organizations that might have an interest in the submitted work.

## Other relationships:

All authors have declared that there are no other relationships or activities that could appear to have influenced the submitted work.

## Conclusion:

Based on the findings of our analysis, we have determined that there is an unambiguous relationship between salivary ferritin and dmft. Children with high dmft scores exhibited a higher salivary ferritin level, and there is no correlation between gender and caries experience.
